# A Publish-Subscribe Model of Genetic Networks

**DOI:** 10.1371/journal.pone.0003245

**Published:** 2008-09-19

**Authors:** Brett Calcott, Duygu Balcan, Paul A. Hohenlohe

**Affiliations:** 1 Philosophy Program, RSSS, Australian National University, Canberra, Australia; 2 Centre for Macroevolution and Macroecology, Australian National University, Canberra, Australia; 3 School of Informatics, Indiana University, Bloomington, Indiana, United States of America; 4 Department of Zoology, Oregon State University, Corvallis, Oregon, United States of America; 5 Center for Ecology and Evolutionary Biology, University of Oregon, Eugene, Oregon, United States of America; Columbia University, United States of America

## Abstract

We present a simple model of genetic regulatory networks in which regulatory connections among genes are mediated by a limited number of signaling molecules. Each gene in our model produces (publishes) a single gene product, which regulates the expression of other genes by binding to regulatory regions that correspond (subscribe) to that product. We explore the consequences of this publish-subscribe model of regulation for the properties of single networks and for the evolution of populations of networks. Degree distributions of randomly constructed networks, particularly multimodal in-degree distributions, which depend on the length of the regulatory sequences and the number of possible gene products, differed from simpler Boolean *NK* models. In simulated evolution of populations of networks, single mutations in regulatory or coding regions resulted in multiple changes in regulatory connections among genes, or alternatively in neutral change that had no effect on phenotype. This resulted in remarkable evolvability in both number and length of attractors, leading to evolved networks far beyond the expectation of these measures based on random distributions. Surprisingly, this rapid evolution was not accompanied by changes in degree distribution; degree distribution in the evolved networks was not substantially different from that of randomly generated networks. The publish-subscribe model also allows exogenous gene products to create an environment, which may be noisy or stable, in which dynamic behavior occurs. In simulations, networks were able to evolve moderate levels of both mutational and environmental robustness.

## Introduction

Models of genetic regulatory networks hold the promise of a deeper understanding of two fundamental processes in biology. First, the relationship between genotype and phenotype in each individual depends on the dynamic behavior of genes interacting with each other and their environment. Second, natural selection acts on the resulting phenotypes produced by this interaction, thus the response to selection and the long-term course of evolution depend on how variation in network properties can be altered by mutation and recombination. Of particular interest is understanding the connection between these two processes, as our assumptions about how these networks are formed affect how they operate *at a time*, and simultaneously how they can change *over time*. As with all modeling efforts, constructing these models requires a balance between simple, general, and easily interpreted models on the one hand, and more complex, specific, and predictive models on the other. Here we present what we call a *publish-subscribe* model of gene regulation. This model adds a layer of complexity to an existing simple model, Kauffman's *NK* networks [1], [2]. Our model produces networks that operate similarly to those in the *NK* model–a number of regulatory genes affect each other, producing a series of activation states that stabilizes to a point or cyclic attractor. What differs is the fashion in which the regulatory connections are made, and hence how they can evolve. The changes we introduce allow for independently mutable regulatory and transcribed regions of a gene, and for regulatory connections to be made via intermediary products. This enables significantly different evolutionary dynamics (for example, significant neutral change can take place) and allows the network dynamics to change in different environments, as the intermediary products can be exogenously introduced. The “environment” of the network may be the external environment or neighboring cells in a multicellular organism. The focal network may also be a module within the total genetic network of an organism [3], in which case its environment includes other components of that larger network. We explore some consequences of these changes for the properties of single networks and the evolution of populations of networks.

The *NK* model has been used to explore the properties and dynamic behavior of genetic networks (e.g. [2], [4], [5]). This model represents a set of *N* genes, where the activation of these genes is represented by a binary state that is expressed (1) or not expressed (0). Each gene is influenced by *K* other genes. Whether or not a gene is expressed at time *t* is decided by a Boolean operation on the previous expression state (at time *t*−1) of the *K* other genes that influence it. In the absence of stochasticity or perturbation, the activation of these *N* genes moves through a series of expression states depending on the initial conditions, ending up in either a stable state or periodic attractor. The entire state space can be described, and each possible attractor enumerated, by starting the network in each of its 2*^N^* possible states and constructing a directed graph in which the nodes are possible states of the network and the edges are transitions among them. These transitions depend only on the connections between the genes and the specific Boolean rules associated with each gene.

The use of discrete, Boolean rules for gene regulation appears justified as a first approximation to data from living organisms [6]–[8]. In a real network, the interactions among genes are mediated by gene products, transcription factors, signaling pathways, cellular machinery, and diffusion processes [9]. In the *NK* network model, all of these processes are collapsed into the edges linking one gene to another. This may be a good assumption in part because biological networks must be somewhat environmentally robust, i.e. buffered against perturbations and stochasticity [10], [11]. This may preclude, for example, dependence on sensitively fine-tuned levels of gene expression. Thus simple *NK* networks seem to capture many of the fundamental dynamics of genetic networks.

However, the assumption of these simple gene-to-gene connections may affect our understanding of the two basic questions raised above. Consider the first issue, the relationship between genotype and phenotype. We wish to know, for example, combinations of parameters for which networks exhibit a certain behavior (e.g. [9]). Randomly generated *NK* networks can provide an estimate, but models including other parts of the genetic regulatory process may widen the volume of parameter space in which solutions are found [12], or change our understanding of the effect on network properties of processes such as gene duplication [13].

Consider also the second issue, the evolution of populations of networks. Evolution is often envisaged by constructing a fitness landscape, a multidimensional surface defined by fitness as a function of genotype (or phenotype), where a single “step” on the surface is equivalent to a one locus mutation of the genotype [14]. Our assessment of the ruggedness of the landscape, and therefore the ability of populations to evolve toward global optima rather than remain on isolated local peaks, depends on the details of the model. In particular, what constitutes a single mutational step determines the structure of variation available to evolution. So we must consider not just how these networks operate, but also how changes in the genotype affect fitness, for this will be crucial to constructing the statistical properties of the fitness landscape. The simplest type of mutation in *NK* networks is the addition or removal of a single connection (or “edge”) between genes in the network (e.g. [2]). Changes in such regulatory influence are often represented as changes to values in a connection matrix (e.g. [9]). Of course, simulated evolution of a population of *NK* networks can proceed by multiple such changes in a single generation or by other types of mutation, such as gene duplication or loss (e.g. [4]), and this has been a productive avenue for research on network evolution. Nonetheless, our view of the landscape of possible network configurations–whether it has a single or multiple adaptive peaks [2] or how connected is the “metagraph” of networks possessing some quality like robustness [8]–depends on which networks are connected to each other by a single mutational step.

In our model, we explicitly consider the process of gene regulation by introducing gene products that mediate the regulatory connections among genes. These gene products may represent proteins, or they may be any of a variety of non-protein regulatory molecules whose role is just beginning to be understood [15]. Each gene is separated into a coding region, which produces a gene product, and a regulatory region, to which gene products may bind. The coding region of each gene acts as a binary switch, either expressed or not in each time step. Whether a gene is expressed–whether the coding region produces its product–depends on the products that are bound to its regulatory region and a set of Boolean rules that translates the binding state of the regulatory region into the expression state of the coding region. The regulatory connections are therefore not specified directly, but rather are an upshot of the correspondence between coding regions and regulatory regions.

For instance, the coding region of a particular gene might produce some product φ. Any gene that has the binding site for φ in its regulatory region will then be regulated by that gene, and also any other gene that produces product φ. This has an effect on the range of variation in network behavior and on what constitutes a single mutational step. We can think of coding regions that contain a conserved DNA motif [16] as transmitting or *publishing* a signal on a certain channel, and regulatory binding sites which bind this motif as *subscribing* on that same channel. If a publisher (coding region) stops transmitting on a channel, then all subscribers (regulatory binding sites) tuned to that channel will be affected. Likewise, if a subscriber is tuned to a channel over which multiple publishers are sending signals, it will be affected by each of these multiple signals. In this way, the equivalent of several connections among genes in the network can be created or destroyed by a single genetic mutation. What constitutes a single step on the adaptive landscape is now significantly different than a model that directly connects or disconnects the regulatory interactions by adding or removing an edge or changing the weight in a connection matrix.

It is worth noting that what we have described here as publish-subscribe has a relevant parallel in the area of modern software construction (indeed, that is where we derived the name) [17], [18]. The move from directly connecting two interacting parts of a software application to connecting them via this more indirect manner has an important result. The two processes are now decoupled, as new upstream processes may influence any processes subscribed to the right message, and likewise new downstream processes can react to a message by simply subscribing to it. This particular kind of “design pattern” [18] ensures that, although the system remains operationally equivalent to one with direct connections, it is far easier to implement changes that re-use the available structure. We might say that implementing the system in this way makes it more “evolvable”, in the sense that modifications are easier to make, and have less chance of having a catastrophic effect. In a similar manner, moving from a model where connections are made directly, to one where the interactions occur indirectly through such a publish-subscribe paradigm, will have important implications in how the system may evolve.

Below we describe the model formally. We derive some basic properties of the structure and dynamic behavior of the networks, both by sampling randomly constructed networks and by analytic means. We then consider how this publish-subscribe view of gene regulatory interactions drives the potential of populations of networks to evolve in response to different regimes of selection.

## Methods

**Figure 1:**
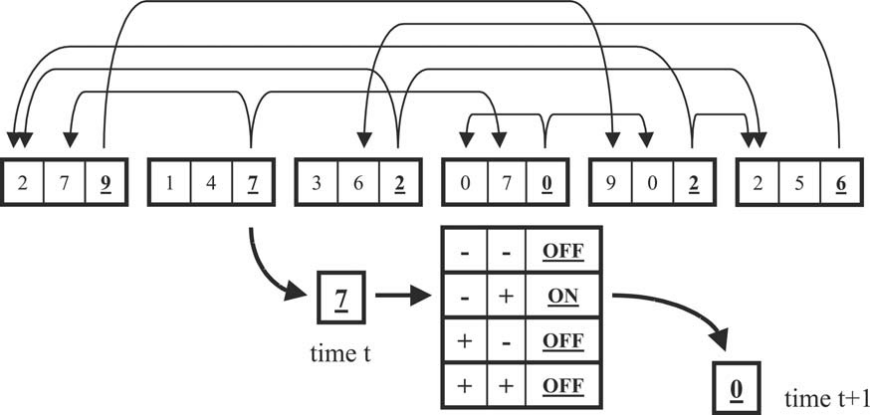
Schematic diagram of the network model. Shown are six genes, each with a regulatory region of length 2 and a coding region (underlined). Arrows represent possible interactions, i.e. directed edges in the network. Below one gene is the Boolean rule set specific to that gene. A “−“ indicates that the binding site is not bound by the corresponding product, and a “+” indicates that it is bound. The gene is then either expressed (“on”) or not (“off”). In this case, if product 7 but not product 0 is present at time *t*, the binding state of the regulatory region of this gene corresponds to the second row of the Boolean table. As a result, the gene is expressed and product 0 is present at time *t*+1. Because 0 occurs in both the regulatory region and the coding region of this gene, it is self-regulating and will not be expressed at time *t*+2.

We consider a conceptually simple model of a genetic regulatory network consisting of *N* genes, each of which includes a regulatory region and a coding region (Fig. 1). The regulatory region consists of a number of binding sites, to which specific gene products may bind. Let us denote the regulatory region of the *i*th gene by ρ*_i_* and its coding region by π*_i_*. We define ρ*_i_* and π*_i_* as sequences of length *l* and 1, respectively:
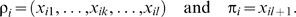
(1)


Each element *x_ik_* (where *k* = 1,…,(*l*+1)) for the sequence of a gene is chosen from an alphabet Π containing *r* letters with uniform probability 1/*r*. So if our alphabet Π = {0,1,2,3}, and length *l* = 3, then one possible gene would be (1,1,3,2). Here ρ = (1,1,3) and π = 2. A network consists of *N* such genes.

Interaction between two genes is mediated by gene products. If the *i*th gene produces a product *x* that matches a binding site in the *j*th gene, then the *i*th gene may regulate the expression of the *j*th gene. We denote the possible interaction (adjacency) matrix by *w*, with elements
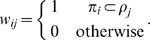
(2)


The in- and out-degree of a gene are calculated by summing the elements of the adjacency matrix, respectively:
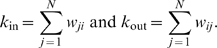
(3)Below we show both numerical and analytic estimations of in- and out-degree distributions [19]. Note that in estimating in- and out-degree distributions, we do not consider the particular set of Boolean rules governing the activity state of each gene. For instance, a given letter *x* may occur in the regulatory sequence of gene *i*. All genes containing *x* in their coding region (i.e., genes that may produce the corresponding product) are considered to be connected to gene *i* in the calculations of degree below. As with *NK* networks, this is the case even if the particular Boolean rules for gene *i* imply that the presence of that product has no effect on the activity state of gene *i*.

In each time step a set of products *R*(*t*) is present, where *R*(*t*)⊂Π. Each binding site in the regulatory region ρ*_i_* is bound if the matching gene product is present (i.e. if *x*_ik_⊂*R*(*t*)). Products are not consumed when they bind; thus the product from a single gene is sufficient for binding the regulatory regions of several genes (effectively, we ignore quantities of gene products). We denote the entire binding state for a gene at time *t* as the vector
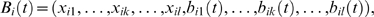
(4)where *b_ik_* denotes the binding state (either bound or not) of the *k*th site in the binding region,
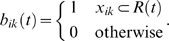
(5)


Note that this state *B_i_*(*t*) may be equivalent to some other binding state *B_j_*(*t*) if the *j*th gene has the same values in its binding region. It may also be equivalent to *B_i_*(*t*−*s*) if the same products were present at time *t*−*s*. This binding state is used to determine whether or not the gene is active, and whether the corresponding value in the coding region will produce a product at time *t*+1.

The value *B_i_*(*t*) locates a unique entry in a Boolean table that returns a value representing whether the corresponding gene is active or not. This table is common to all genes in the network (and all networks if there is a population of networks evolving). We will denote the table as Ψ. This table contains all possible combinations of values in a binding region and their possible bound state. Providing a global table of each particular Boolean response to a combination of bound products provides a realistic degree of stability to the system: two genes with identical regulatory regions presented with the same set of intermediary products will always do the same thing. The activity state of gene *i* at the following time step is read from this table as
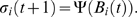
(6)


The activity state σ*_i_* is binary, taking values of either 1 or 0. If σ*_i_*(*t*+1) = 1, the product *x_i_* will be produced by gene *i*, so that *x_i_*⊂*R*(*t*+1). If σ*_i_*(*t*+1) = 0, then *x_i_* will not be produced by gene *i*, but the identical product *x* may be produced by another gene. The activation state of the network at time *t* is given by Σ(*t*) = (σ*_1_*(*t*),…, σ*_N_*(*t*)). In constructing the table Ψ, the value 1 is assigned to each σ*_i_* with probability *p*, so that *p* gives a measure of the overall probability of gene activity.

**Figure 2:**
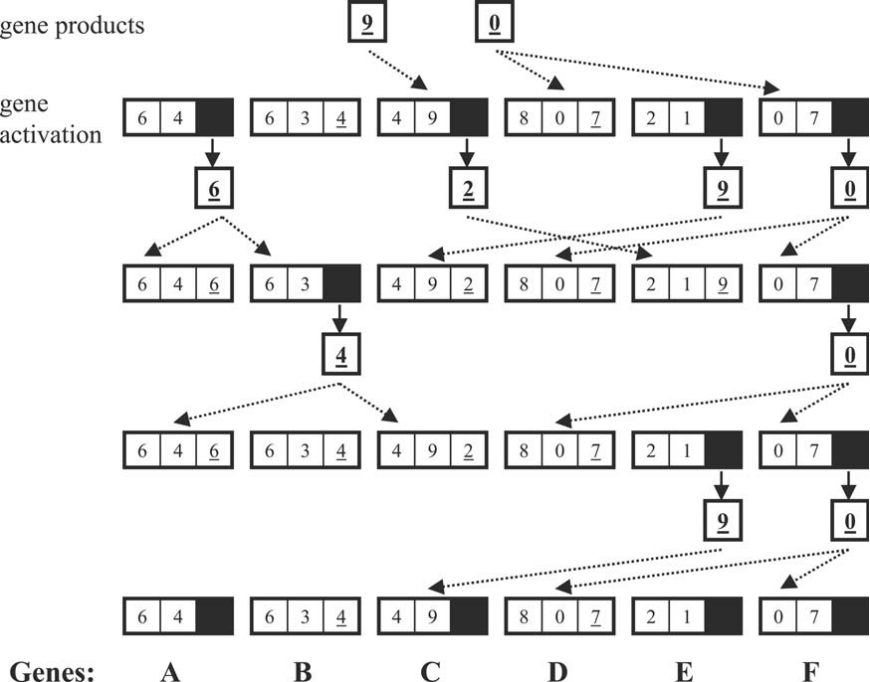
Diagram of four time steps in a 6-gene network. In the initial conditions, products 9 and 0 are present. Filled boxes represent expressed genes, dotted arrows represent binding of products to regulatory regions, and solid arrows represent production of gene products. From these initial conditions, this network enters a stable period-3 cyclic attractor. Boolean tables are not shown.

This model results in several possible regulatory patterns: for instance, multiple genes with the same product have identical regulatory effects, genes may regulate themselves (e.g. genes A and F in Fig. 2), and products can have either inhibitory or activating effects (e.g. the effect of product 6 on gene A versus gene B in Fig. 2). Because there is a finite number of genes and gene activity is binary, there is a finite number of states of the network. Therefore, given a set of starting conditions and no stochasticity, the network reaches a stable attractor. The attractor can be a single state (i.e. the same set of gene products in each time step) or a cycle (the same sets of products produced at regular intervals). Fig. 2 illustrates a period-3 attractor over the entire network, with some genes (D and F) in a stable state.

Because each gene's activation σ*_i_*(*t*) is binary, the dynamics of any particular model network is much like the *NK* model, in that many binary states determine a single downstream gene's state by a set of Boolean operations. What differs is how the regulatory connections are constructed, and thus how they might evolve. How we initiate the network also differs. Rather than setting it into a particular state, its initial conditions are defined by the introduction of an initial set of products *R*(0). Note that this means that, although there are 2*^N^* possible states of the network, not all of these states may be strictly reachable. There may be no combination of products that can produce a particular activation state Σ. In the course of simulations, we may activate any state and see what products it produces. But driving the dynamics of the network purely by introducing gene products already places a constraint on possible states that the network can enter. Finally, mediating connections among genes by using gene products means that a network can operate in an “environment” of exogenous gene products that influence its dynamic behavior. This environment may be stable or temporally variable, as we illustrate below.

## Results

### Basic Properties of the Network

The interactions between a set of genes in the model described above can be represented as a directed graph, where the nodes represent genes and the edges represent connections among genes in the publish-subscribe model. The edges are directed because of the way we define the regulatory and coding regions of our genes. For instance, the product *x_i_* of gene *i* may affect the activity state of gene *j* at the next time step, but not vice versa. Thus each gene may affect “downstream” genes and simultaneously be affected by “upstream” genes. The number of upstream and downstream genes connected to a particular gene is the in-degree and out-degree (respectively) of that gene. Each network can be characterized by its in- and out-degree distribution–the frequency distribution of in- and out-degree across all genes, or nodes in the network.

Degree distributions are important indicators of the organizational principles underlying networks and have been the focus of network theory approaches to gene regulation. The in- and out-degree distributions of real transcriptional regulatory networks exhibit different functional forms. In-degree typically displays an exponential decay and is restricted to a narrow interval, while the out-degree distribution typically has a broad tail [20]–[25]. It has been shown [25] that in- and out-degree distributions together are sufficient to reproduce most of the global topological properties of genetic regulatory networks such as degree-degree correlation [26] and clustering coefficient [27]. Degree distributions are also considered to be important in determining the resistance of networks to perturbations (robustness) and the ability of populations of networks to evolve (evolvability) [5]. With these motivations, here we derive the directed degree distributions to provide better insight on the properties of our model networks. We have calculated these distributions both numerically and analytically. Numerical results were calculated from frequency distributions of a large number of networks, each generated by randomly and independently assigning letters from the alphabet Π to each regulatory and coding site, while keeping the alphabet size, regulatory region length, and total network size constant. Below we present results for relatively small values of alphabet size (*r* = 10), regulatory region size (*l* = 3), and total network size (*N*). These parameter values, particularly alphabet size and total network size, are likely to be much smaller than those measured in actual genetic networks [16], [21], [28], but they provide a starting point for exploring the behavior of the publish-subscribe model. Our goal is to compare our results to the more basic *NK* model, so that our conclusions can be tied to the addition of explicit gene products. Because of the modularity found in empirical gene networks, we can envision smaller networks as modules operating in the context of a larger organismal network; in this context, the “environment” of exogenous gene products that we consider below represents other interacting modules of the overall network.

**Figure 3:**
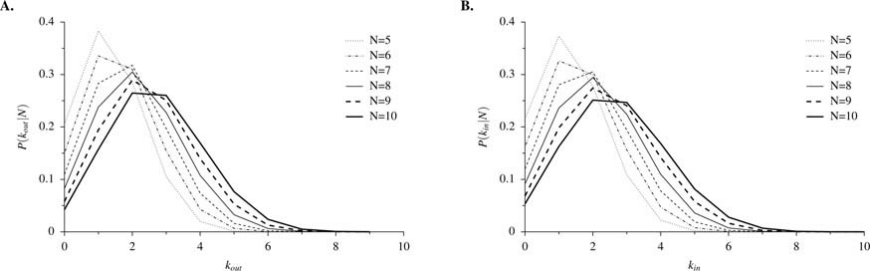
Degree distributions for small networks. (A) Out-degree and (B) in-degree distributions are shown for networks of size *N* = 5 to *N* = 10. Each distribution is constructed from 10^6^ independent, randomly generated networks with parameter values *r* = 10 and *l* = 3.

**Figure 4:**
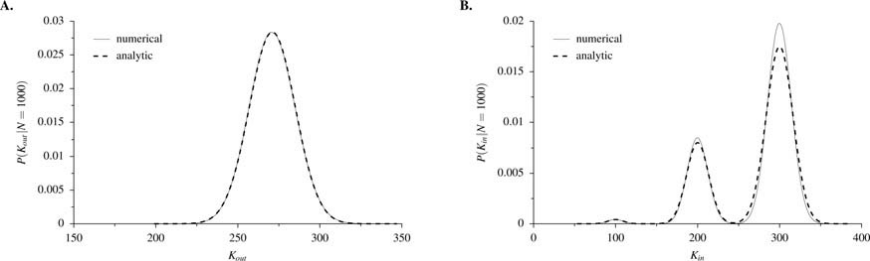
Numerical and analytic degree distributions for large networks. (A) Out-degree and (B) in-degree distributions for networks of size *N* = 1000. Numerical distributions are constructed from 10^6^ independent, randomly generated networks with parameter values *r* = 10 and *l* = 3.

For relatively small values of *N*, both in-degree and out-degree distributions shift to the right as *N* increases (Fig. 3). In other words, as *N* increases, the number of genes with products corresponding to a binding site of gene *i* increases (in-degree), and the number of genes with binding sites corresponding to the product of gene *i* increases (out-degree). To explore the large-*N* limit, Fig. 4 shows in-degree and out-degree distributions for large networks (*N* = 1000). In the large-*N* limit, such that all sequences of length *l* are likely to be realized, the out-degree distribution approaches a single binomial distribution (Fig. 4A). In contrast, the in-degree distribution approaches a superposition of binomial distributions, with separate peaks corresponding to the number of different letters contained in a sequence of length *l* = 3 randomly sampled with replacement from the finite alphabet Π (Fig. 4B). For example, the smallest peak in Fig. 4B is the result of genes whose three binding sites contain the same letter *x*, and the largest peak is the result of genes with a different letter at each of the three binding sites.

To calculate the out-degree distribution analytically, first we determine the probability of finding a given letter *x* in a randomly chosen sequence of length *l*, which is given by
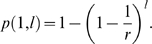
(7)This equals the probability of the product of gene *i* occuring in the regulatory sequence of gene *j*. Thus in the large-*N* limit, out-degree *k_out_* is binomially distributed:
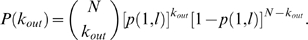
(8a)The mean and variance of this distribution are given by
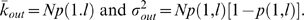
(8b)This analytic solution for out-degree distribution closely matches the numerical estimate (Fig. 4A).

An analytic solution for the in-degree distribution is more complex, being in fact a superposition of binomial distributions. This is because a regulatory sequence of length *l*, chosen from a finite alphabet of size *r*, may contain duplicate letters. Let *I* be the number of different letters *x* occurring in a regulatory sequence, so that 1≤*I*≤min(*l*,*r*), and let ω(*I*) be the number of possible sequences containing exactly *I* different letters *x*. The total number of possible regulatory sequences is 
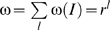
. The value ω(*I*) can be directly calculated in terms of the parameters *r* and *l*. Denote the multiplicity of letter *x_i_* in a sequence of length *l* by *n*(*x_i_*). Given *I* and *l* there are two constraints on *n*(*x_i_*):
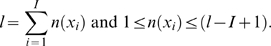
(9)For a set of *I* different letters with multiplicities {*n*(*x_i_*)}, the number of possible sequences is a multinomial coefficient
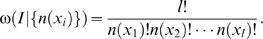
(10)Combining equations (9) and (10) we get the number of regulatory sequences containing exactly *I* different letters:
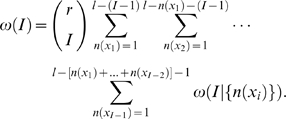
(11)If we sum over the multiplicities in equation (11), we get
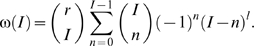
(12)Note that ω(*I*) also gives us the number of possible tuples *B_i_*(*t*) in the table Ψ:
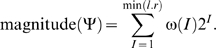
(13)For regulatory sequences with *I* different letters, the in-degree distribution is
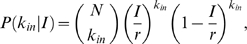
(14a)where *I*/*r* is the probability that a randomly selected gene product *x* matches one of the *I* different letters in the regulatory sequence. The mean and variance of this distribution are
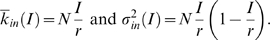
(14b)The total in-degree distribution is thus:
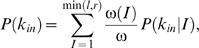
(15)where ω(*I*)/ω is the probability that a randomly selected regulatory sequence contains *I* different letters. This analytical solution closely matches the numerical estimate (Fig. 4B).

### State Space

Although a graph representing the regulatory interactions between genes tells us something about the structure of possible interactions in the network, the full dynamics of a particular network–what that network does–can be represented by exploring its state space. A network activation state space contains all possible activation states that the network can take, and the transitions between each of them.

**Figure 5:**
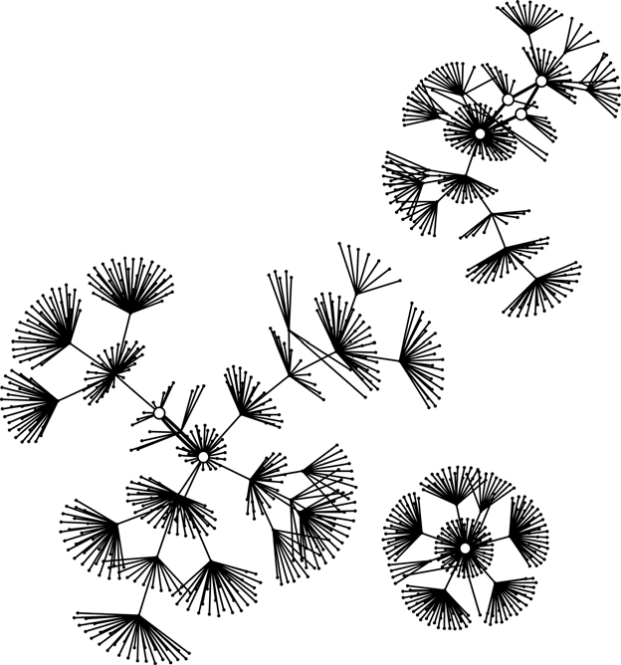
State space of a randomly generated network. The state space of a network can be represented as a directed graph. Each point (node) represents an expression state of the network, and lines (edges) connecting them represent transitions from one time step to the next. This network has *N* = 10 genes, and therefore 1024 states. The network has three attractors (open circles), of which one is a single steady state where an identical set of gene products is present at each time step, and the other two are cyclic attractors of period 2 and 4, respectively.

For a given number of genes *N*, there is a total of Ω = 2*^N^* possible activation states of the network. For a finite network size *N*, the state space is also finite. Starting from an initial state, the system will eventually return to a previously visited state. Thereafter it will follow stable or cyclic behavior, if no stochasticity or exogenous gene products are introduced. The set of states that constitutes a cycle is called an *attractor*, and the number of states it contains is the *attractor length*. All the states converging to an attractor constitute its *basin of attraction*, and the number of states in a basin of attraction is the *basin size*. The state space of a network can be represented as a graph (Fig. 5), just as the possible regulatory links among genes can be. But these two graphs are very different things. For example, the in-degree of a gene is the number of other genes that may regulate it; the in-degree of a particular state of the network is the number of states at time *t* that will end up at that state at time *t*+1. We call the in-degree of a network state the *precursor number* of that state. Below we consider these characteristics of the state space of networks of size *N* = 10.

**Figure 6:**
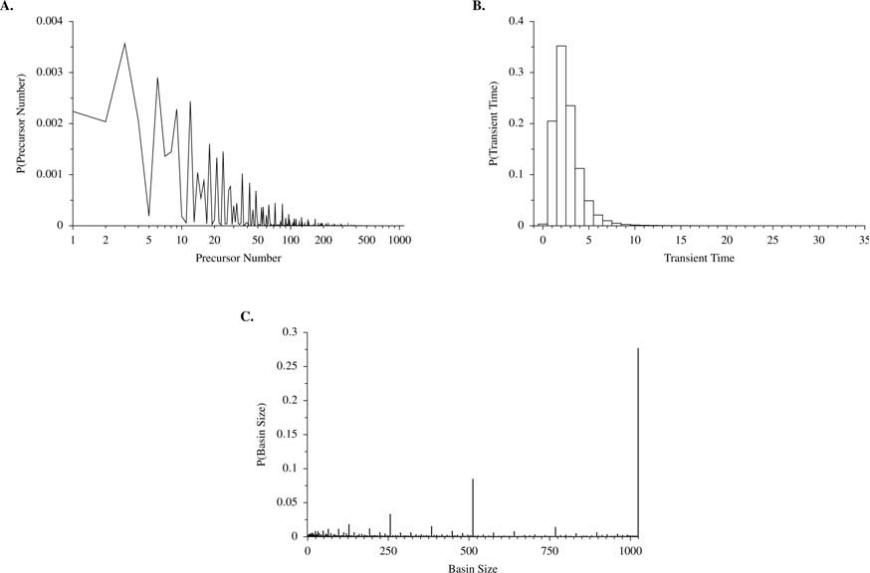
Precursor number, transient time, and basin size for networks of size *N* = 10. (A) Frequency distribution of precursor number across network states, estimated from 40,000 randomly generated networks, on a log scale. Note that *P*(0)≈0.96 has been suppressed, meaning that the large majority of states have no precursor. (B) Frequency distribution of transient time, estimated from 40,000 randomly generated networks. The maximum value of τ is 31 in this number of realizations. (C) Frequency distribution of attractor basin size, defined as the number of states that lead to a given attractor, estimated from 40,000 randomly generated networks of size *N* = 10. Note the peaks at Ω/2*^n^*, *n* = 0,1,2,…, where Ω = 1024 is the total number of states in each network.

In a randomly constructed network, the vast majority of network states have no precursor (Fig. 5). Such states are unreachable by the network, unless they are used to initiate the network in a simulation. An immediate consequence of this fact is that the average *transient time* that it takes to reach an attractor starting from an arbitrary state is very short compared to the state space size Ω. To make these statements clear we have calculated the probabilities *P_p_*(*n_p_*) and *P_τ_*(τ) that an arbitrary state has *n_p_* precursors and transient time τ, respectively. These quantities are displayed in Fig. 6 for a network of size *N* = 10. It should be noted that *P_p_*(0) increases as *N* increases (not shown) and *P_p_*(*n_p_*) may have any value between 0 and Ω. Note also that the mode of the transient time distribution shifts to the right as *N* increases (not shown).

We consider also the basin size distribution, *P_s_*(*n_s_*), which is the probability of having a basin of attraction of size *n_s_* (Fig. 6C). *P_s_*(*n_s_*) is concentrated on values *n_s_* = Ω/2^m^, *m* = 0,1,…,∞, and decreases dramatically as *m*→∞. This means that in an arbitrary realization we may observe only the peaks at *n_s_* = Ω or *n_s_* = Ω/2. The case of *n_s_* = Ω corresponds to a network with a single attractor whose basin of attraction encompasses the entire network. This pattern is similar to that found in *NK* networks when *K* is relatively small (e.g., *K* = 1) [29].

**Figure 7:**
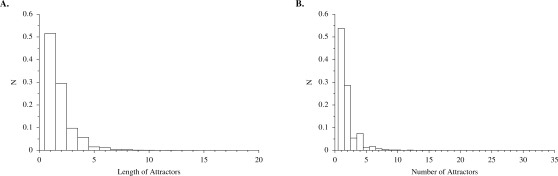
Length and number of attractors in networks of size *N* = 10. (A) Frequency distribution of length of attractors, estimated from 40,000 randomly generated networks. The maximum attractor length in this sample was 31. (B) Frequency distribution of the number of attractors in each network, estimated from 80,000 randomly generated networks. The maximum number of attractors was 17 in this sample.

**Figure 8:**
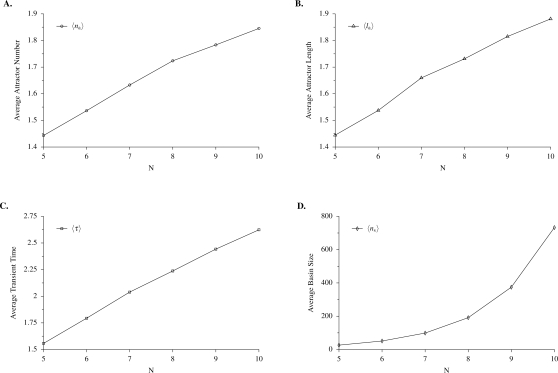
Attractor properties as a function of network size in small networks. The average attractor number (A), attractor length (B), and transient time (C) increase linearly as a function of network size *N*, while average basin size (D) increases exponentially.

Fig. 7 shows the distribution of number of attractors, *P_a_*(*n_a_*), and the probability that a given attractor has length *l_a_*, *P_l_*(*l_a_*), in randomly constructed networks of size *N* = 10. Note that *P_a_*(1) and *P_l_*(1) decrease as *N* increases (not shown). Below we evolve populations of networks using selection on attractor number and attractor length. The distributions shown in Fig. 7 for randomly constructed networks illustrate the range of variation in these properties available to evolution in a randomly generated population, and they provide a benchmark against which to measure the efficacy of evolution to find relatively small regions of network space where fitness is maximized. Fig. 8 shows the mean values for attractor number (*n_a_*), attractor length (*l_a_*), transient time (*τ*), and attractor basin size (*n_s_*), over a range of small values of *N*. Note that the first three of these measures increase roughly linearly with *N*, while basin size increases exponentially. Thus basin size increases roughly proportional to state space size Ω, which itself is an exponential function of *N*. It had been believed that the average number of attractors of *NK* networks increased as the square root of system size [29], but recent numerical studies [30] have shown that this quantity increases linearly with *N*, as it does in our model.

### Evolution of the Networks

In this section we use simulations to explore what sort of networks can be produced by selecting for a particular property in a population of networks. In the following simulations we restricted the changes to point mutations (changes in single letters in either the regulatory or coding regions of genes), and modeled the evolution of an asexual population. The model could also be extended to include recombination among genomes, and other types of mutations such as gene duplications and deletions (e.g. [13]), but we leave this for a later time. To begin, we selected on two network properties: attractor length and number of attractors. Given that attractors form the basis of any subsequent control of gene expression, it is important to show the lability these properties have under a simple selective regime. Such network traits may also relate to fitness in biological systems by corresponding to the identity and behavior of different cell types in multicellular organisms [2], or alternative states of a genetic network module [31]. Here they provide a simple first test of how the networks might evolve, and the resulting evolved networks provide an interesting comparison with the randomly sampled networks studied above.

**Figure 9:**
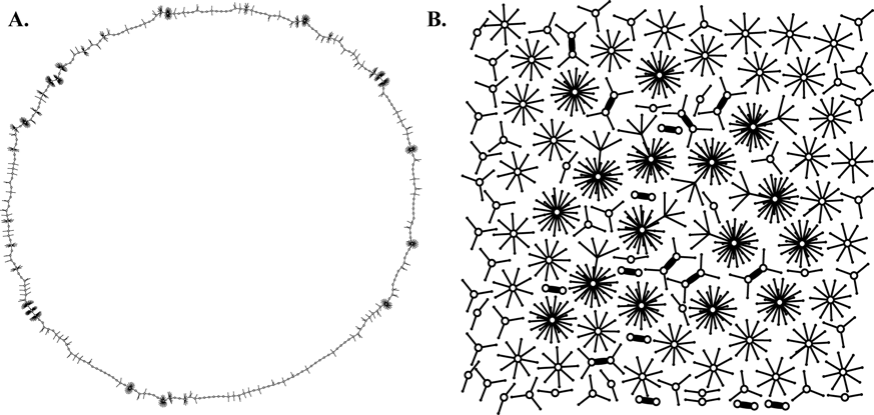
State spaces of evolved networks. (A) State space of a network evolved in a population of 100 networks after 100 generations of selection for large attractor size. The attractor shown has length 254. Here *N* = 10, *l* = 3, and *p* = 0.5. (B) State space of a network evolved under selection for many attractors. This network has 112 attractors. All other parameter values as in (A).

In both cases we generated an initial population of 100 networks, analyzed the state space of each network and assigned it a fitness equal to either the number of states in the largest attractor or the total number of attractors in the state space. We then generated a new, non-overlapping generation of 100 networks. Each network in the new generation was produced, without recombination, from a single parent drawn randomly from the previous generation. The probability that a network was selected as a parent was directly proportional to its fitness. Each reproductive event included a single random point mutation in the network's genome, with each site in either regulatory or coding regions having an equal probability of mutation. We repeated this procedure for 100 generations. The state space of the fittest networks resulting from selection for attractor size and attractor number are shown in Fig. 9.

Selecting on these particular properties resulted in some very atypical networks. The results of these simulations were strikingly different from randomly generated networks, such as those depicted in [2] or in Fig. 5. For instance, the maximum attractor length in a sample of 40,000 randomly generated networks was 31 (Fig. 7A). In contrast, simulated evolution was able to produce an attractor length of 254 in less than 100 generations. Similarly, selection for attractor number produced a network with 112 attractors, far greater than the maximum of 17 in the sample of 40,000 networks shown in Fig. 7B. The large number of possible graphs in this network model means that random sampling to estimate distributions of network properties may fail to capture evolutionarily important parts of the space of all possible networks. Furthermore, it appears that such atypical networks can reliably be reached in relatively few generations, even when the range of variation available to selection is constrained to single point mutations as it was in these simulations.

**Figure 10:**
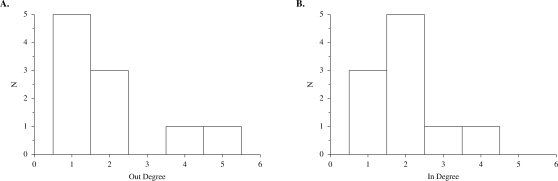
In- and out-degree distributions of evolved networks. Shown are (A) in-degree and (B) out-degree distributions for the evolved network in Fig. 9A, the result of selection on attractor size, and (C) in-degree and (D) out-degree distributions for the network in Fig. 9B, the result of selection on attractor number. These distributions may be compared to the random expectations for *N* = 10 in Fig. 3.

**Figure 11:**
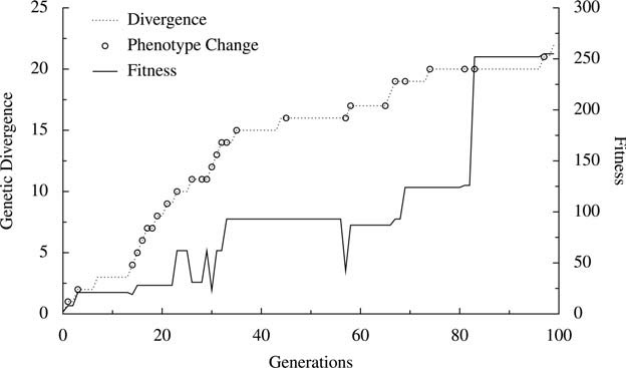
Genotype, phenotype, and fitness in a single evolving lineage. Shown are three network properties over 100 generations in the lineage leading to the network shown in Fig. 9A. Genotypic divergence (dotted line) is the number of letters *x* in the network sequence, either in regulatory or coding regions, different from the ancestor. Changes in phenotype (open circles) are points at which the attractors of the network change, whether or not this results in a change in length of the longest attractor. Fitness (solid line) is the length of the longest attractor in the network state space. Note that changes in either genotype or phenotype may be effectively neutral, without corresponding changes in fitness, and that large changes in fitness can occur with relatively small changes in genotype.

Surprisingly, despite their rapid evolution in the character subject to selection (attractor length and number, respectively), these evolved networks did not seem atypical in other respects. Their in-degree and out-degree distributions, shown in Fig. 10, were very close to the expectation for randomly generated networks of their size (*N* = 10; Fig. 3). The dramatic changes in attractor length and number were not the result of concomitant changes in degree distribution. This independence of network properties is further illustrated in Fig. 11. Fitness did not increase smoothly, but rather made occasional large jumps. In contrast, genotypic change occurred more steadily over the course of the simulation. Many genetic changes were neutral with respect to attractor length. In addition, length or number of the other attractors in the network's state space changed without affecting length of the longest attractor; these are phenotypic changes that were also neutral with respect to fitness. Neither genotypic change nor change in other phenotypic traits was a reliable predictor of change in fitness in these simulations, despite the relative simplicity of the trait being selected.

### Evolution in an Environment

We have been treating networks as though they operated in isolation, subject only to the gene products produced by the network itself. Because intermediary products control the activation of genes in our model, the introduction of any exogenous products can influence the downstream activation and resultant attractor of the network. This gives our model an important additional property over the *NK* model: the state space, including the number and type of attractors, is a property of a particular network *combined with* a particular environment.

**Figure 12:**
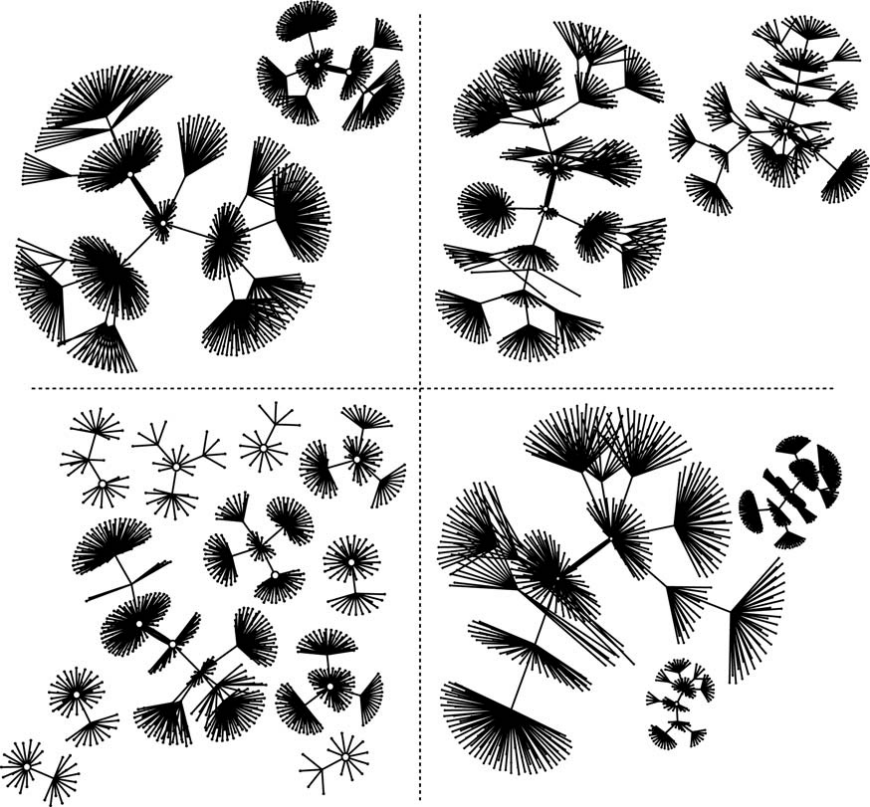
State space of a single network subject to different environmental conditions. Shown are the state spaces of a single network of size *N* = 10 under four different environments. Each environment represents a different set of gene products that are constantly present (e.g. exogenously produced) and available to bind to regulatory regions in the network. Note that a single network can vary in both the number and size of attractors depending on the environment.

A network with no exogenous input has a single state space. However, if we assume that our environment provides a constant set of products, not produced by the focal network itself, but still able to bind and regulate the functioning of the network, the state space for any single network now depends on the particular environment of exogenous products in which the network operates (Fig. 12). Under constant environmental conditions the network will settle into one attractor, depending on the starting point. When environmental conditions change, the state that was previously in an attractor may shift to the edge of a basin, and the network may move to a new state. The introduction or removal of different products can have many effects on the state space, such as changing the number of attractors, the size of their basins, or the set of expression states contained in their basins. The maximum number of possible environments is 2*^r^*, where *r* is the number of possible letters in the alphabet *Π*. Thus the number of state space graphs corresponding to a single network may be as large as 2*^r^*.

One property of a network is the degree to which these state spaces are similar, or fall into broad groups. This similarity may be considered a measure of the environmental robustness of the network. If the network continues to act relatively unchanged (the attractors remain constant) in various environments (differing exogenous inputs), then the network operation is robust to these changes. Although robustness in Boolean networks can be thought of in this way, our model permits us to explore a much more dynamic sense of robustness (in contrast with [9], for example). The environments in which genetic networks operate are both sources of noise and sources of important signals, either from the external environment, from other parts of a multicellular organism, or from other modules in the organism's overall genetic network [31]. Fitness depends on responding appropriately to the signals and ignoring the noise. Viewed in this way, what must be robust is the *reaction norm* of the network–its ability to react in a plastic and appropriate manner under various environments by distinguishing signal from noise.

We simulated evolution in a series of simple environments, in which fitness was determined by their ability to respond “appropriately.” If some *indicator* product was present in the environment, a network had high fitness if it produced some other *functional* product. If another indicator product was present, the network was fit if it produced a second, different, functional product. A network had high fitness by doing the right thing at the right time: in environment *A*, produce product *a*, and in environment *B*, produce product *b*. Doing the right thing implies not doing the wrong thing also–producing product *b* in environment *A* reduced fitness, and a network that simply produced *a* and *b* constitutively did not have high fitness. We selected on networks' ability to respond correctly to two different environments that alternated over time.

We evolved a population of 100 networks of size *N* = 10. Each network was exposed to the first environment for 10 time steps, and then switched to the second environment for another 10 time steps. The networks were then returned to the original environment. This environment switching continued until the network had been exposed to each environment 5 times. Fitness was calculated as the number of correct functional products produced, minus the number of incorrect functional products, summed across all time steps. Gene products that were not the functional product in either environment did not affect fitness.

In addition to this alternation of environmental signals, we tested the ability of networks to evolve robustness to environmental noise. In the stable environment, the only exogenous products were the indicator products. In this simulation the evolved networks quickly behaved exactly as required, changing their required output in the presence of different indicator products. In the noisy environment, the indicator product was present with 2 other products, randomly chosen at each time step. Achieving a high fitness under noisy conditions was more difficult to evolve, and the networks remained at lower fitnesses throughout the simulation under noisy conditions. However, we found that a network that had evolved in a noisy environment would often perform perfectly in a stable environment.

**Figure 13:**
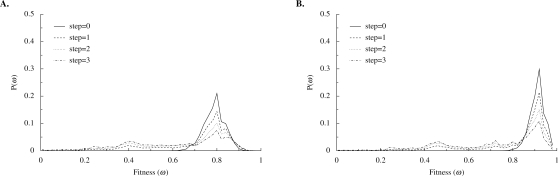
Fitness of evolved networks in noisy environments. Frequency distributions of fitness for single evolved networks subjected to 10,000 trials in a noisy environment. Solid lines indicate the fitness of the evolved network, while dashed lines indicate the fitness of networks that are 1, 2, or 3 mutational steps away from the evolved network. (A) Fitness of the network produced by evolution in a stable environment. (B) Fitness of the network produced by evolution in a noisy environment.

What sort of difference is there between a network evolved in a stable environment and one evolved in a noisy environment? We tested this by subjecting the fittest network from each simulation to a number of trials (10,000) in a noisy environment. The sample distributions generated are shown in Fig. 13. We assessed both the original network (steps = 0) and a sample of 1-, 2-, and 3-step mutants from this network. This gives us some idea of the fitness of the networks in the local mutational neighborhood, and thus an indication of the ruggedness of the fitness landscape close to the peak on which the evolved network sits. Evolving the networks in a noisy environment did indeed produce a more consistently environmentally robust network, shown by both the relative positions and the widths of the peaks in the frequency distributions in Fig. 13. The decline in fitness with increasing numbers of mutations away from the original network is similar for the networks evolved in both stable and noisy environments. Thus these networks have roughly equivalent mutational robustness. In terms of the fitness landscape, the fitness peaks to which the networks have evolved in both stable and noisy environments are somewhat intermediate between broad plateaus and precipitous spires, which would allow for some near-neutral variation to persist in mutation/selection balance.

## Discussion

Simple models of genetic networks have led to general conclusions about the properties of network architecture and how they affect network evolution [1], [2], [4], [5], [32]. At the same time, a growing number of technological and analytical tools allow the direct measurement of regulatory networks in natural systems [33]–[39], so that a number of empirical networks have been described in detail [21], [28], [40], [41]. In seeking to connect these growing fields, modeling efforts can proceed by adding layers of complexity and assessing the degree to which features of the model better approximate empirical results. Here we have added a degree of complexity to simple *NK* networks, using a publish-subscribe view of gene regulation. Although our model shares some basic similarities with the *NK* model, we have found some tantalizing differences in both the properties of single networks and in the evolution of populations of networks.

First, the pattern of degree distributions from randomly constructed networks in our model is substantially different from that of previous models. In Kauffman's [1] original *NK* model, each gene has exactly *K* inputs and in-degree distribution is therefore a Dirac delta function. In randomly constructed networks under the “standard” *NK* model [4], regulatory inputs to each gene are assigned independently with a given probability, resulting in unimodal binomial (or equivalently for large *N*, Poisson) distributions for in- and out-degree. In scale-free networks, in-degree distribution follows a power law *P*(*k*)∼*k* ^−γ^ while out-degree follows a Poisson distribution, or vice versa [4]. In contrast, our publish-subscribe model produces an in-degree distribution that is multimodal due to the superposition of binomial distributions with different mean values. The fact that in-degree and out-degree distributions differ in form from each other in our model also contrasts with the standard *NK* model. This qualitatively different pattern is a consequence of the matching rule between the different nodes, i.e. between the coding and regulatory sequences. Thus, although the networks in our model exhibit similar dynamics to those of Boolean *NK* networks, the distributions of basic network properties differ as a result of the publish-subscribe regulatory framework. A network model based on a similar matching rule was able to reproduce global topological properties of the yeast gene regulatory network [25]. These properties include not only degree distributions, but also other network descriptors such as clustering coefficient, rich-club coefficient, degree-degree correlation, and *k*-core decomposition.

This divergence from previous models is echoed as well by the networks evolved in our simulations. Generally, degree distribution is believed to be a central feature of a network and a key predictor of its dynamic behavior in other respects [5]. For example, the importance of scale-free degree distributions for other properties like robustness and evolvability has been established in several studies of *NK* networks [4], [42]. However, in our publish-subscribe model, it appears that dynamic behavior may be to some extent uncoupled from degree distribution. In the simulations above, attractor length and number evolved far outside the distribution expected from randomly generated networks, but degree distribution remained remarkably similar to random. The degree distributions of the evolved networks give us no clue to the general principles by which length and number of attractors may evolve. Conversely, degree distribution may be a poor predictor of other network properties in this model. Other topological properties (e.g. [25]) may be more relevant to the evolutionary dynamics of our publish-subscribe model, and this issue should be explored further. However, additional metrics that are directed toward specific tasks, such as robustness to various types of change, may be necessary to fully compare across networks and predict evolutionary dynamics.

In the broader context of dynamic behavior and evolution of genetic regulatory network models, two issues have received particular attention: evolvability and robustness. A critical component of evolvability is the presence of neutral variation in a population [43]–[46]. Evolution in our network model produces neutral variation in genotype, as seen in Fig. 11, that has no immediate effect on either phenotype or fitness. From an adaptive landscape perspective, this neutral change can be seen as meanderings along neutral ridges in the landscape [47], [48]. The importance of this neutral variation is its effect on the fitness of subsequent mutations. In our model, as in natural systems [49], genes often interact epistatically, so that the fitness effect of a single mutation depends on the allelic states of other loci. Thus the genetic background against which a mutation arises may determine whether it is favored by selection, and therefore whether it sweeps to fixation and increases the average fitness of the population as a whole. Neutral mutations change the genetic background that determines both the sign and the magnitude of the fitness effects of subsequent mutations.

Our network model illustrates the mechanism of neutral variation in the publish-subscribe view of gene regulation. For example, regulatory binding sites may mutate to a state for which there is not currently a matching gene product being produced. At the time, this mutation may be neutral, with no effect on the phenotype of the network. However, this mutation has created a new subscriber, ready to receive a signal from a publisher, or coding region. Such a mutation in a publisher may occur in the future, and thus a new connection is made between two genes. In addition, the number of transcriptional regulators (gene products) is limited in our model [15], [50]. As a result, multiple neutral mutations in the form of publishers (or subscribers) tuned to the same signal can accumulate as neutral changes with no effect on fitness. When a single mutation in a subscriber (or publisher) shifts to the matching signal, multiple new connections are formed. The effect on phenotype, and perhaps fitness, as a result of this single mutation is magnified by the presence of existing variation. In fact the ability of mutations to have broader effects on phenotype in this way may be an important component of evolvability [51].

In our simulations we explored the evolution of environmental robustness, which is the ability of a network to perform (i.e., maintain high fitness) in the face of a noisy environment. Incorporating the ability for networks to react to the local environment enables us to explore a number of possibilities. Here, we have emphasized that robustness can be a dynamic, rather than a static, property of networks. The publish-subscribe model allows us to evolve networks whose reaction norm is robust under noisy environments. The shift from a static to a dynamic conception of robustness may have important implications. Consider an idea introduced by Kauffman [2], in which the attractors in genetic networks are viewed as analogous to cell types in a multicellular organism [52]. For the *NK* model, the attractor into which a network falls is fixed for a particular genetic network and the starting conditions. In multicellular development, however, the environment is, in part, other cells, and the process of differentiation may be driven by dynamic interactions between cells rather than the isolated properties of a single cell [53]. The evolution of this plastic response to the local cellular environment, and the evolution of its subsequent robustness, may be a key element in understanding the emergence of multicellularity [54]. Alternatively, the focal network may be a module of a larger genetic network, and organismal fitness may depend on the network's ability to respond appropriately to signals from other modules.

A large number of issues could be explored further with the publish-subscribe model. First, in our estimates of degree distribution, we considered two genes to be connected if the coding region of one gene matched a site in the regulatory region of the other. However, this ignores the particular Boolean rules of expression for the second gene, whose expression state may not actually depend on the first gene's product; in fact, whether this dependence is present may itself depend epistatically on the expression states of yet other genes [2]. Calculation of degree distribution in this expanded sense soon gets quite complicated, although it may be necessary for more direct comparisons to empirical data, such as gene co-expression networks or expression time series [34].

Second, we assumed here that a single coding region produces a sufficient concentration of gene product to bind any number of matching regulatory sites. The consequences of this assumption, or alternatively of competition among binding sites for limited gene product copies, could be explored further. Relaxing this assumption would not change the observed patterns of degree distribution of networks, according to the rules by which we calculated it. However, it would introduce an element of stochasticity into the activation of genes at each time step if single gene products were to bind to either one or another regulatory site with some probability between 0 and 1. As a result, our conception of the state space of a network would also change. Under the current assumption, the out-degree of any node in the state space network is one, but relaxing this assumption would produce some states with probabilistic edges connecting to multiple other states. This would result in an additional concept of robustness that could be explored: the robustness of attractors to stochastic shifts outside of their attractor basin as a result of the stochastic binding of gene products.

Third, one could explore the consequences of variation in several of the parameters. Our goal here was to explore the properties of the simplest publish-subscribe model, so in our evolutionary simulations we held alphabet size, regulatory region length, and total network size constant. Varying these parameters across networks may have implications for measures of network topology and for the evolutionary dynamics of populations of networks. Regulatory region length could also vary across nodes within a network; in a network model similar to ours such variation produced similar qualitative behavior but improved the fit to empirical data on topological descriptors from yeast networks [25]. Change in this parameter has also been implicated in the evolution of organismal complexity [55]. Among other effects, longer regulatory regions would provide a larger mutational target for regulatory versus coding regions. It remains an outstanding question to what extent changes in regulatory versus coding regions play different roles in phenotypic evolution [56], [57], and the publish-subscribe model explicitly separates the two. We plan to address this issue in future work. In our simulations, we used networks of relatively small size (*N* = 10), which can be thought of as modules within a larger network. However, simulations of larger networks, particularly in the noisy or fluctuating environments that we described, could be used to address the evolution of modularity itself; that is, do networks evolve some degree of internal separation of components that partition the response to environmental signals? Alternatively, can the behavior of larger networks be adequately represented by studies of smaller networks?

Finally, we addressed network evolution solely in the context of single-step mutations. The publish-subscribe model could easily be extended to address other types of mutations, such as gain or loss of binding sites in regulatory regions, gene duplication and divergence [13], or whole genome duplication. Nonetheless, our results suggest that the publish-subscribe model holds remarkable evolutionary potential even when mutation is restricted to single steps.

Our publish-subscribe model of genetic regulatory networks adds a layer of complexity to the common *NK* networks by making the gene regulation process more explicit, and by using a rule system for matching gene products to regulatory sites that affect the expression state of other genes. In this way it is similar to yet more complex models. Examples include the Artificial Genome class of models [58]–[61], which create an information sequence analogous to DNA, and content-based networks [19], [25], [62], where the focus is on the topological properties of the networks rather than their dynamics. The production of new, more complex, variants on well-studied models in biology can often aid in two ways. First, the introduction of new parameters might suggest that there is behavior outside the scope of the simpler model. Second, the introduction might allow us to ask different questions. The publish-subscribe model appears to do both.
